# Design Low Crosstalk Ring-Slot Array Structure for Label-Free Multiplexed Sensing

**DOI:** 10.3390/s140915658

**Published:** 2014-08-25

**Authors:** Lijun Huang, Huiping Tian, Jian Zhou, Yuefeng Ji

**Affiliations:** 1 State Key Laboratory of Information Photonics and Optical Communications, School of Information and Communication Engineering, Beijing University of Posts and Telecommunications, Beijing 100876, China; E-Mails: hljnet@163.com (L.H.); zhoujian19890109@sina.com (J.Z.); 2 Department of Physics and Information Engineering, Huaihua University, Huaihua 418008, China

**Keywords:** integrated optical devices, optical sensing and sensors, low crosstalk, photonic crystals

## Abstract

We theoretically demonstrate a low crosstalk ring-slot array structure used for label-free multiplexed sensing. The proposed sensors array is based on an array of three ring-slot and input/output line defect coupling waveguides. Each ring-slot cavity has slightly different cavity spacing and different resonant frequency. Results obtained using two dimensional finite-difference time-domain (2D-FDTD) simulation indicate that the resonant frequencies of each sensor unit in response to the refractive index variations are independent. The refractive index sensitivity is 134 ∼ 145.5 nm/RIU (refractive index unit) and the *Q* factors more than 10^4^ can be achieved. The calculated detect limit lower than 1.13 × 10^−4^ RIU is obtained. In addition, an extremely small crosstalk lower than −25.8 dB is achieved among the array of three ring-slot cavities. The results demonstrate that this multiplexed sensor array is a promising platform for integrated optical devices and enables highly parallel label-free detection.

## 1. Introduction

Integrated optical devices based on photonic crystals (PhCs) are a promising platform for high density integration of devices which significantly enhance light-matter interaction [1–3]. In recent years, a wide variety of applications have been proposed and demonstrated, including optical switches [4,5], light emitting diodes [6], lasers [7,8], all-optical logic gates [9,10], filters [11,12], power splitters [13,14] and highly efficient modulators [15–17]. In particular, PhC microcavities of various architectures have attracted significant interests in molecule sensing due to their compact size, low detect limit (DL) and high sensitivity (*S*) [18–24], thus far, 1D PhC [20,25,26], 2D PhC [27–29], L3 cavities [30], slot waveguides [24], ring cavities [31–33], have been studied widely for sensing, since the size of PhC sensors are approximately three orders of magnitude less than commercial integrated-optic sensors [34]. For these devices, in a solid medium, and liquid samples in close proximity to the cavities' surface, light can be used to detect the alteration of refractive index caused by the evanescent tails of the guided mode inside the cavities. For use as a label-free sensor, higher refractive index (RI) molecules are bound to the sensor surface, superseding the lower refractive index water, and the resonant wavelength shifts as the refractive index of surrounding cavities changes, that is, the surrounding index can be detected by measuring the wavelength shift via a spectrometer. However, most of these designs concentrate on a single sensor and the target numbers of samples probed at one time are relatively small.

Next generation biosensor platforms must have inherently high degrees of multiplexing capabilities [3]. The more the number of disease markers which can be probed, the more information that can be obtained. Multiplexing capability for pathogen detection is required not only to screen against the large numbers of different pathogens which can be differentiated in a given sample but also to provide specific subtype information. For example, subtype information is extremely important in virology for tracking emerging viruses and designing appropriate vaccines [35]. Therefore, PhC array sensors recently have been proposed and demonstrated to overcome the insufficiency and realize multiple biosensing. Zlatanovic *et al.* [36] described time-resolved label-free monitoring of protein binding in a physiological buffer using a photonic crystal microcavity sensor of 50 μm^2^ total area with an effective detection area of 0.272 μm^2^, however, at a concentration of 0.67 nM of probe biomolecules with K_d_ ≈ 6 × 10^−7^ M, the resonant wavelength shift is less than 0.05 nm with a resonant quality (*Q*) factor of ∼300. Mandal *et al.* [25] demonstrated a nanoscale optofluidic sensor array based on a silicon waveguide with a 1D PhC microcavity for label-free detection of biomolecular interactions in aqueous environments, in which the device exhibits a bulk refractive index sensitivity of 130 nm/RIU and a *Q* factor of around 3000. In addition, the single notch extinction ratio is only 4 ∼ 10 dB. Yang *et al.* [37] studied theoretically a nanoscale photonic crystal sensor array on monolithic substrates using a side-coupled resonant cavity array, in which the sensitivity of this device is 115.60 nm/RIU and a *Q* factor is less than 3000 on monolithic substrates. Yang *et al.* [38] investigated theoretically a nanoscale low crosstalk photonic crystal integrated sensor array. The proposed device consists of array of side-coupled PhC resonant cavities with *Q* factors over 2 × 10^3^, in which sensitivities of ∼100 nm/RIU and crosstalk lower than −4 dB are observed. Moreover, the sensing signal of each cavity may interact with each other because of the crosstalk of multi-cavity parallel sensing when the number of sensors integrated is increased.

This paper mainly introduces a label-free multiplexed sensor array with very low crosstalk by designing a ring-slot array structure. The proposed device consists of an array of three ring-slot cavities with a high *Q*-factor over 10^4^. By adjusting the width of the ring-slot, each cavity has different cavity spacing and different resonant peaks, and the resonant peaks are shifted independently when the refractive index surrounding the cavity is altered. By using FDTD calculation, considering the RI of ambient functionalized area varied from 1.330 to 1.377, the RI sensitivity (*S*) is 134 ∼ 145.5 nm/RIU, simultaneously the calculated detect limit (DL) lower than 1.13 × 10^−4^ RIU is obtained. In addition, we further study the crosstalk among three sensors when the RI of a sensor is varied and the other two sensors are not, and the maximum crosstalk among three sensors is less than −25.8 dB, and the crosstalk is, to the best of our knowledge, the lowest ever reported [38]. The results show that this multiplexed sensing array is a promising platform for integrated optical devices and enables highly parallel detection with very low crosstalk.

## 2. PhC Integrated Ring-Slot Sensors Array Design

**Figure 1.**
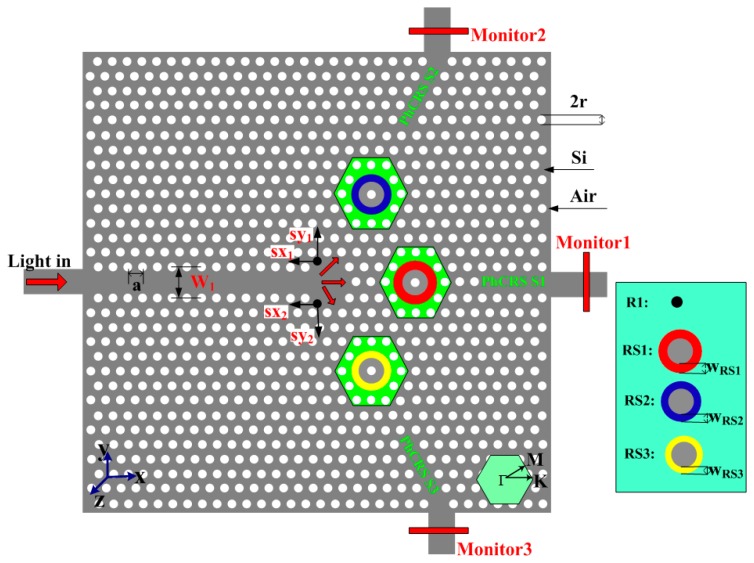
The schematic of nanoscale integrated PhC ring-slot sensors array (PhCRSSA). The air holes and ring-slot in green area served as functionalized area.

The nanoscale integrated PhC ring-slot sensor array (PhCRSSA) is shown in Figure 1. As seen in this figure, triangular lattice air holes are arranged in silicon (n_si_ = 3.48). The proposed PhCRSSA consists of three ring-slot cavities (PhCRSS1, PhCRSS2, PhCRSS3), in which six air holes are removed and etched a ring-slot in the center of structure and light is coupled input and output in each ring-slot cavity through a line defect waveguide. We simulate the PhCRSSA structure by using the open source FDTD software Meep which further divides space into a discrete grid and the fields are evolved in time using discrete time steps [39]. The grid and the time steps are made finer and finer, and it becomes a closer and closer approximation for the true continuous equations. During the simulation process, the TE Gaussian-pulse source is used and run for several iterations. The resolution is set to 20 (that is, with a grid spacing of *a*/20, where *a* represents the lattice constant). One-spatial unit thick perfectly matched layer (PML) which surrounds the simulated area absorbs the fields leaving the simulated region to implement reflections. The light source is placed at the head of the input line defect waveguide and the monitor is placed at the end of three output line defect waveguides. By dividing the output power detected with the monitor by the input power of the source, we obtain the transmittance spectra. The lattice constant *a* equals to 378 nm and the air holes radius *r* is 0.34*a* (128.5 nm). In order to obtain slightly different resonant ring-slot cavity spacing and different resonant peaks, the width of each ring-slot in PhCRSSA is designed to have slightly different cavity spacing. As seen in Figure 1, the specific parameters of PhCRSSA are as follows: ${\text{W}}_{1}=\sqrt{3}a$; PhCRSS1: W_RS1_ = 0.24*a*; PhCRSS2: W_RS2_ = 0.22*a*; PhCRSS3: W_RS1_ = 0.20*a*. To enhance the transmission efficiencies in the PhCRSS2 and PhCRSS3, based on our experience of adjusting structure and similar to former solution [40], we further alter the position of black air holes and achieve optimized results. The distances of black air holes moved are as follows: sx_1_ = 0.17*a*, sy_1_ = 0.17*a*, sx_2_ = 0.17*a*, sy_2_ = 0.17*a*. The air holes and ring-slot of each ring-slot resonant cavity in green shadow area are served as functionalized area, that is, the RI of air holes and ring-slot in this area is changed in order to investigate the sensitivity and crosstalk of the array of three sensors.

**Figure 2.**
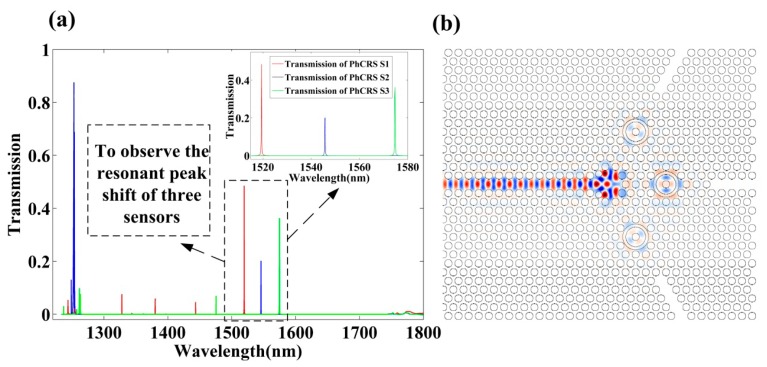
(**a**) The transmission spectra of three ring-slot cavities (PhCRSS1, PhCRSS2, PhCRSS3) in water environment (RI = 1.330), and observing the resonant peaks of three ring-slot cavities surrounded by black dashed line shift when the RI of functionalized area is changed; (**b**) The steady-state electric field profile through three ring-slot cavities.

The output transmission spectra obtained for the optimal three ring-slot cavities are shown in Figure 2a, and the steady-state electric field profile is shown in Figure 2b. As seen in Figure 2a, the transmission efficiencies have response peaks at 1519.44 nm, 1545.72 nm, 1574.65 nm in a water environment (RI = 1.330) with respect to the red solid line, blue solid line, green solid line, respectively, and the *Q* factors of the three peaks are equal to 11,029, 10,153, 10,669, respectively, and three peaks surrounded by black dashed line are chosen to observe the resonant wavelength shift of integrated three sensors when the RI in the functionalized area is varied.

## 3. The Properties Analysis of Integrated PhC Ring-Slot Sensors Array

**Figure 3.**
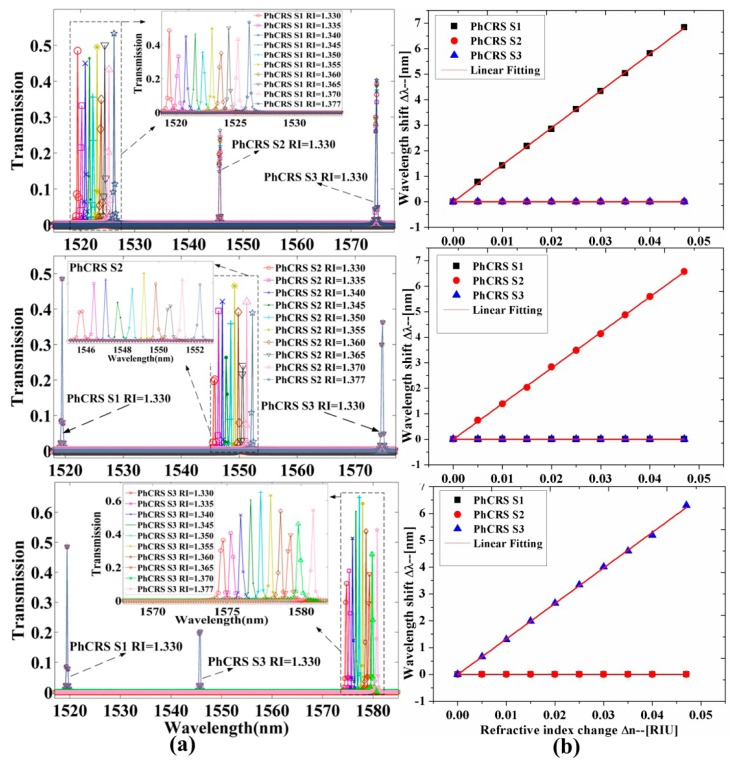
(**a**) The transmission spectra of three ring-slot cavities (PhCRSS1, PhCRSS2, PhCRSS3) when the RI of functionalized area of one sensor is changed and the others are not; (**b**) Wavelength shift Δλ as a function of the RI of functionalized area increasing.

To analyze the properties of the integrated PhC ring-slot sensor array in aqueous solution to implement biosensing, the RI of the functionalized area of three sensors is changed, respectively. Figure 3a shows the transmission spectra of three ring-slot cavities (PhCRSS1, PhCRSS2, PhCRSS3) when the RI of functionalized area of one sensor is changed and the others aren't. As shown in Figure 3a, the upper row shows the transmission spectra and the resonant peaks are red-shifted (longer wavelength) when the RI of functionalized area of PhCRSS1 is changed and the others aren't, and the inset shows the amplified image of the transmission spectra of PhCRSS1 when the RI of functionalized area of PhCRSS1 is changed by 1.330, 1.335, 1.340, 1.345, 1.3501, 1.355, 1.360, 1.365, 1.370, 1.377, respectively. *Q* factors of more than 10^4^ are achieved for these resonant peaks, and this is higher than ever reported [25,36,37]. We can also find that the transmission spectra of PhCRSS2 and PhCRSS3 are barely transformed when the RI of PhCRSS1 is changed. Similarly, the middle row shows the transmission red-shifted spectra of PhCRSS2 when the RI of PhCRSS2 is transformed, and the lower row shows the red-shifted transmission spectra of PhCRSS3 when the RI of PhCRSS3 is changed.

The resonant wavelength shift of each ring-slot cavity (PhCRSS1, PhCRSS2, PhCRSS3) as a function of the RI variations of functionalized area is shown in Figure 3b. As seen in this figure, the resonant wavelength shift of each ring-slot cavity is fitted linearly when the RI of functionalized area is varied (RI = 1.330, 1.335, 1.340, 1.345, 1.350, 1.355, 1.360, 1.365, 1.370, 1.377, respectively). The sensitivities *S* (S = Δλ/Δn) of the three sensors *S_1_* = 145.5 nm/RIU, *S_2_* = 140 nm/RIU, *S_3_* = 134 nm/RIU, are observed, respectively. In order to analyze quantitatively the properties of the sensor array, the sensitivity (S) and the resolution (R) are combined to calculate the detection limit (DL). The DL describes the minimum detectable limit and the sensor resolution (R) characterizes the smallest possible spectral shift that can be accurately measured. In practice, the resolution (R) depends on the experimental noise level, and one-tenth of line-width can easily be resolved in [30]. Here we assume a minimal resolvable wavelength shift to a line-width R given by [18,30]:

(1) $$R=\frac{\lambda_{0}}{10\cdot Q}$$

where λ_0_ represents the resonant wavelength, therefore, the detection limit can be expressed by [18]:

(2) $$\text{DL}=\frac{R}{S}=\frac{\lambda_{0}}{10\cdot Q\cdot S}$$

Based on Equation (2), we can calculate that the detectable minimum change in refractive index is lower than 1.13 × 10^−4^ RIU. References [41] and [42] show the relation that the rate of change with glucose concentration was assumed to be 0.0014 g/100 mL (or per 1% concentration) at temperatures of 20 °C, 25 °C and 30 °C. In other words, the RI of a glucose solution is changed from 1.3340 to 1.3480 when the concentration of glucose changes in the range from 0% to 10% at 30 °C temperature, and the variation span lies in the region from 1.330 to 1.350, This means that the proposed sensor array in theory can be integrated with a fluid flow network to probe glucose molecules.

## 4. Crosstalk Analysis of the Integrated PhC Ring-Slot Sensors Array

The crosstalk of each channel is an important index for an integrated PhC ring-slot sensor array to implement biosensing. Ideally, what is needed is an integrated sensor that enables highly parallel detection of biomolecule interactions with a high area density of independent sensors that can operate without crosstalk [43]. To evaluate the crosstalk degree of each sensor unit, the maximum crosstalk (MCT) is defined as follows: [38]

(3) $$\text{MCT}=10\cdot \mathrm{lg}\frac{T}{T_{i}}$$

where *T_i_* represents the minimum transmission efficiency of other sensors at the resonant frequency ω_0_, and *T* represents the maximum transmission efficiency of one sensor with the same frequency ω_0_ when the functionalized area RI of this sensor is varied. The calculated MCT between each sensor is shown in Table 1.

As shown in Table 1, the maximum crosstalk of S1-S2 and S1-S3 are both increased with the increase of functionalized area RI of PhCRSS1, because the resonant peak in Figure 3a is shifted to a longer wavelength and close to the resonant peaks of PhCRSS2 and PhCRSS3 when the functionalized area RI of PhCRSS1 is increased, whereas the crosstalk of S1-S3 is less evident than S1-S2, since the interval of the resonant frequencies of S1-S3 is larger than S1-S2 and the minimum transmission efficiency of PhCRSS3 is higher than PhCRSS2. With the increase of functionalized area RI of PhCRSS2, the crosstalk of S2-S1 slowly become smaller and the crosstalk of S2-S3 become larger, because the resonant peak is red-shifted, resulting in the resonant peak being far away from the resonant peak of PhCRSS1 and approaching the resonant peak of PhCRSS3. The same variation tendency is suitable for the crosstalk of S3-S1 and S3-S2 with the increase of functionalized area RI of PhCRSS3. An evident from the results seen in Table 1, an extremely low crosstalk between each other adjacent sensor units in the integrated PhC ring-slot sensor array (lower than −25.8 dB) is observed, and this is, to the best of our knowledge, less than ever reported [38]. In theory, for a PhCs-based sensor array structure, the primary source of crosstalk comes from individual sensors as well as nonlinear effects, including stimulated Raman scattering, and Kerr-polarization dependent loss [44–46]. In practice, the free spectrum range, the position of the resonant peak and quality factor are affected due to fabrication imperfections and noise levels in optical sensors [47]. Consequently, the crosstalk among individual sensor should be increased. Generally, the value of crosstalk is more than the theoretical value. Therefore, this low crosstalk feature could be potentially very useful for monolithically integrated and multiplexed sensing.

## 5. Conclusions

We have theoretically designed and demonstrated a label-free integrated ring-slot sensor array structure to implement multiplexed sensing. Different resonant peaks of the array of three ring-slot sensors are obtained by adjusting the width of three ring-slots. Simulation results showed that a *Q* factor of more than 10^4^ and sensitivity (*S*) 134 ∼ 145.5 nm/RIU are achieved when the functionalized area RI is from 1.330 to 1.377, and simultaneously a detection limit (DL) of less than 1.13 × 10^−4^ RIU is obtained. In addition, an extremely low crosstalk of less than −25.8 dB is achieved among the three sensors. The results show that the proposed nanoscale integrated PhC ring-slot sensors array is a promising platform for monolithically integrated and multiplexed sensing.

## Figures and Tables

**Table 1.** The maximum crosstalk (dB) between the proposed ring-slot sensors array (PhCRSS1, PhCRSS2, PhCRSS3).

RI		1.330	1.335	1.340	1.345	1.350	1.355	1.360	1.365	1.370	1.377

Crosstalk(dB)											

Sensors											
Changing	S1-S2	−33.0	−32.7	−32.2	−31.5	−30.9	−30.4	−30.0	−29.2	−28.7	−27.8
PhCRSS1 RI	S1-S3	−41.0	−40.9	−40.5	−40.2	−39.7	−39.5	−39.0	−38.6	−38.5	−37.0

Changing	S2-S1	−25.8	−25.9	−26.0	−26.2	−26.3	−26.4	−26.5	−26.5	−26.6	−26.7
PhCRSS2 RI	S2-S3	−50.4	−51.4	−50.4	−48.7	−47.5	−46.8	−46.5	−46.7	−46.9	−46.0

Changing	S3-S1	−28.0	−28.0	−28.0	−27.9	−28.0	−28.0	−28.0	−28.0	−28.1	−28.1
PhCRSS3 RI	S3-S2	−52.1	−52.2	−51.4	−51.8	−52.4	−52.1	−52.6	−52.9	−52.8	−52.5
